# Pathophysiological Defects and Transcriptional Profiling in the *RBM20^-/-^* Rat Model

**DOI:** 10.1371/journal.pone.0084281

**Published:** 2013-12-19

**Authors:** Wei Guo, Jonathan M. Pleitner, Kurt W. Saupe, Marion L. Greaser

**Affiliations:** 1 Muscle Biology Laboratory, University of Wisconsin-Madison, Madison, Wisconsin, United States of America; 2 Department of Medicine, University of Wisconsin-Madison, Madison, Wisconsin, United States of America; Temple University, United States of America

## Abstract

Our recent study indicated that RNA binding motif 20 (Rbm20) alters splicing of titin and other genes. The current goals were to understand how the Rbm20^-/-^ rat is related to physiological, structural, and molecular changes leading to heart failure. We quantitatively and qualitatively compared the expression of titin isoforms between Rbm20^-/-^ and wild type rats by real time RT-PCR and SDS agarose electrophoresis. Isoform changes were linked to alterations in transcription as opposed to translation of titin messages. Reduced time to exhaustion with running in knockout rats also suggested a lower maximal cardiac output or decreased skeletal muscle performance. Electron microscopic observations of the left ventricle from knockout animals showed abnormal myofibril arrangement, Z line streaming, and lipofuscin deposits. Mutant skeletal muscle ultrastructure appeared normal. The results suggest that splicing alterations in Rbm20^-/-^ rats resulted in pathogenic changes in physiology and cardiac ultrastructure. Secondary changes were observed in message levels for many genes whose splicing was not directly affected. Gene and protein expression data indicated the activation of pathophysiological and muscle stress-activated pathways. These data provide new insights on Rbm20 function and how its malfunction leads to cardiomyopathy.

## Introduction

Recently our group cloned a novel splicing factor, RNA binding motif 20 (Rbm20), using a natural mutant rat and found that Rbm20 regulates titin isoform transition and the splicing of 30 other genes [1–3]. Rbm20 is one of a series of 48 putative RNA binding proteins that have been identified genomically by the presence of an RNA binding motif. Many of these proteins have been shown to be involved in RNA alternative splicing. The relationship of RBM20 mutations to human dilated cardiomyopathy was first reported by Brauch and coworkers [4]. They found five different mutations in eight families with cardiac enlargement (determined by echocardiography) and reduced ejection fraction. Li and coworkers [5] described an additional 4 mutations in the same gene. All are point mutations and 7 of the 9 are localized in the RS (arginine-serine) domain. Affected individuals often die suddenly in young adulthood (20s to 40s), and they display a varied degree of cardiac fibrosis and arrhythmia. Heart transplantion may be necessary in affected individuals [3–5]. RBM20 mutations may account for as many as 3% of dilated cardiomyopathy patients [6]. 

Titin is found in highest concentrations in heart and skeletal muscle. The single mammalian gene is expressed in multiple isoforms as a result of alternative splicing [7]. There are two major classes of cardiac titin isoforms: N2B and N2BA [8]. Before birth, mammalian heart mainly expresses the more compliant N2BA isoforms. During the perinatal period the larger N2BA (~3.7 MDa) isoform is gradually replaced by the smaller N2B (~3.0 MDa), with the latter becoming the predominant titin isoform in rat adult left ventricles (LV) [9–11]. An intermediate sized N2BA isoform (~ 3.4 MDa) constitutes an increased proportion of the titin in the adult hearts of larger mammals, including humans [12,13]. Interestingly, titin splicing appears to be absent in the Rbm20^-/-^ rat, and a larger titin isoform N2BA (~3.8MDa) is expressed almost exclusively throughout life. 

 A number of disease states (dilated cardiomyopathy, ischemia, hypertrophic cardiomyopathy) have been linked to changes in titin splicing. Several studies have shown that the alteration of the ratios of the two isoforms is related to cardiomyopathic passive stiffness and human heart disease [14–16]. More recent analyses showed that left ventricle biopsies from patients with diastolic heart failure (HF) had a reduced N2BA/N2B titin ratio [17]. Chronically ischemic LVs of coronary-artery-disease (CAD) patients with congestive heart failure (HF) had nearly 50% N2BA titin (compared to N2BA+N2B) while approximate 30% N2BA was found in the LVs of control donor patients [18]. Analysis of explanted nonischaemic human DCM hearts again demonstrated increased proportions of N2BA/N2B and decreased cardiomyopathy passive stiffness [19]. Long-term hypothyroidism (which results in diastolic dysfunction) changed the titin isoform ratios as well. Propylthiouracil (PTU) treatment in rats induced the expression of additional cardiac PEVK and Ig domain exons similar to those in the large titin isoform of the fetal heart. Consequently, titin-based passive and restoring forces were found to be significantly reduced in cardiac muscle of PTU-treated rats [20–22]. It is clear that titin-isoform switching adjusts myocardial passive stiffness, but how the higher or lower proportions of titin isoforms relates to heart disease remains to be characterized. 

The current study with the Rbm20^-/-^ rat shows that protein isoform transition of titin is consistent with transcript levels of titin, and altered splicing due to the deficiency of Rbm20 in rats is also associated with abnormal cardiac structure, physiology, and transcriptome. Expression of many heart-failure related genes was altered even though there were no direct changes in their splicing. The complex collaboration of multiple gene regulation, growth factor, and hormone related protein synthesis pathways in these rats provide a novel model for the human RBM20 related cardiac pathology. This report characterizes the RBM20^-/-^ rat as an aid to better understand the human physiological and molecular phenotype.

## Materials and Methods

This study was carried out in strict accordance with the recommendations in the Guide for the Care and Use of Laboratory Animals of the National Institutes of Health. The protocol was approved by the Animal Use and Care Committee of the University of Wisconsin-Madison (protocol M01715).

### Animals and sample preparation

The study was performed with the Rbm20^-/-^ rat [1–3]. Rats used in the current work were crosses of Sprague-Dawley (SD) and Fisher (F) 344 (SDF) [(SDxF344)F1-Rbm20^m1Mlgw^ ] or SD X F X Brown Norway (BN) (SDFBN) [(SDxF344)F1xBN-Rbm20^m1Mlgw^ ] (all strains were originally obtained from Harlan Sprague Dawley, Indianapolis, IN). Wild type animals [(SDxF344)F1-Rbm20^m1Mlgw+/+^][(SDxF344)F1xBN-Rbm20^m1Mlgw+/+^] with the same genetic backgrounds were used for all comparisons. Animals were maintained on standard rodent chow. This study was carried out in strict accordance with the recommendations in the Guide for the Care and Use of Laboratory Animals of the National Institutes of Health. The protocol was approved by the Animal Use and Care Committee of the University of Wisconsin-Madison (protocol M01715). Hearts were removed immediately after euthanasia and the left ventricle, right ventricle, and atria were separated. Samples were obtained from animals ranging in age from 16 days fetal to 500 days after birth. Tissues were snap-frozen in liquid nitrogen and stored in a -80°C freezer prior to protein gel sample and RNA preparation. 

### Agarose gel electrophoresis

Titin isoforms were resolved using a vertical sodium dodecyl sulfate (SDS)-1% agarose gel electrophoresis (VAGE) system [16]. Protein samples of left ventricle were prepared with urea-thiourea buffer (8M urea, 2M thiourea, 75mM DTT, 3% SDS, 0.05% bromophenol blue, 0.05M Tris, pH 6.8) from wild type, and RBM20^-/-^ homozygous rats. Density analysis of titin bands was performed using NIH Image to determine isoform ratios. Myosin heavy chain proportions were determined by the method of Warren and Greaser [23]. Coomassie stained gels were analyzed by the method of Mitov and Campbell [24]. 

### Exercise training and measurement of exercise capacity

Eight Rbm20^-/-^ homozygous rats (4 males and 4 females) and 10 wild type rats (6 males and 4 females) with ages of 6 to 7 month were used. Rat exercise capacity determination was adapted from methods used previously [25]. Rats were acclimated to the treadmill by walking at a speed of 16 m/min, 5 min/d, for 2 weeks on a 4-lane Columbus Instruments treadmill. After this acclimatization period, rats in each age group were then assigned to training at a speed of 20 m/min, 10 min/d, for another two weeks. At the end of the 4-week period, maximal exercise capacity was measured twice for each rat in tests separated by 2 days. The protocol for the maximal exercise capacity test consisted of walking at 14m/min for 5 minutes followed by 2 m/min increases in speed every 2 minutes until the rat reached exhaustion. Rats were considered exhausted when they failed to stay off of a shock bar. All the tests were conducted by the same individual.

### Light and electron microscopy

Wild type (n=3) and homozygote mutant (n=3) adult rats (1-1.5 years of age) were anesthetized and maintained on isoflurane. Heparin (2000 IU units per kg body weight) was injected intraperitoneally. After 10 minutes, the chest cavity was opened and 3M KCl was injected into the left ventricle to arrest beating. The aorta was cut, and a cannula attached for connection to a Langendorf perfusion apparatus. Blood was washed out by perfusing with 20-30 ml of high potassium Ringers solution (60 mM NaCl – 60 mM KCl – 1.2 mM NaH_2_PO_4_ - 25 mM HEPES – 11 mM glucose – 1.2 mM MgCl_2_, pH 7.2). The heart was subsequently perfused with Karnovsky fixative (2% paraformaldehyde – 2.5% glutaraldehyde in 0.1 M phosphate buffer, pH 7.2) (80-90 ml at a flow rate of about 10 ml/min). The left ventricle free wall was cut into 2 X 1 X 1 mm pieces and transferred to fresh fixative for 3 hr at room temperature. After several rinses with buffer, the tissue pieces were fixed for 1 hr in 1% osmium tetroxide in 0.1 M phosphate buffer, rinsed in water, and dehydrated in an ethanol series to 70% ethanol. Samples were stained in block with 5% uranyl acetate in 70% ethanol for 1 hr. They were subsequently transferred to 100% ethanol, then a 50:50 ethanol:propylene oxide mixture, and then into 100% propylene oxide. Samples were transferred into 25% Epon-Araldite resin - 75% propylene oxide for 1 hour, then in 50% resin for 3 hours, in 75% resin for 4 hours, and in 100% resin in a vacuum oven overnight at room temperature. Samples were cured in a vacuum oven for 48 hours at 60°C. Thin sections of 60-70 nm were obtained and stained with lead citrate and uranyl acetate. Digital images were obtained using a Philips CM 120 transmission electron microscope. Sections were obtained from 5 to 10 different blocks for each animal.

Skeletal muscle electron microscopy was conducted using the same animals described above. Small fiber bundles from the tibialis anterior, longissimus dorsi, soleus, and psoas major were tied to wooden dowels and immersed in the same composition paraformaldehyde-glutaraldehyde fixative used for cardiac muscle samples. The remaining preparation methods were identical to those described above. Sections from approximately 10 different regions of each fiber bundle preparation were observed and digitally recorded.

### Gene expression analysis

Total RNA was extracted from left ventricle (~50mg) of three wild types and three Rbm20^-/-^ homozygous rats at day 49 with 1ml TRIzol reagent (Invitrogen) separately and further purified with RNeasy columns (Qiagen) according to the manufacturer’s protocols. RNA concentration was measured with a NanoDrop ND 1000 spectrometer (NanoDrop Technologies, Wilmington, DE), and RNA integrity was assessed on an Agilent 2100 Bioanalyzer (Agilent Technologies, Palo Alto, CA). Double stranded cDNA was synthesized from total RNA (SuperScript II system; Invitrogen). An *in vitro* transcription reaction was then performed to obtain biotin-labeled cRNA from the double-stranded cDNA (Enzo BioArray High Yield RNA Transcript Labeling kit; Enzo Diagnostics, Farmingdale, NY). The cRNA was fragmented before hybridization, and then mixed in a hybridization mixture containing probe array controls, BSA, and herring sperm DNA. A cleanup procedure was performed on the hybridization cocktail using an RNeasy spin column (Qiagen), after which it was applied to the Affymetrix Rat 230 2.0 probe array. Three wild type (Wt) and three Rbm20^-/-^ homozygote (Hm) rats (age 49 days) were analyzed with 6 Affymetrix GeneChip Rat Genome 230 2.0 arrays. Hybridization was allowed to continue for 16 h at 45°C in a GeneChip 640 hybridization oven, after which the arrays were washed and stained with phycoerythrin-conjugated streptavidin (Molecular Probes, Eugene, OR). Images were scanned using a GeneArray scanner (Agilent Technologies, Palo Alto, CA). 

Chip quality and hybridization experiments were assessed by methods previously described [26]. After passing the quality control, GeneChip raw data were subsequently processed by the log scale robust multi-array analysis (RMA) method [27]. After RMA normalization, one way ANOVA was performed to detect expression differences in allele status and a Student t-test with a p value cut-off of 0.01 and a minimum 1.5-fold change between two specified genotype groups was used to identify genes that were significantly regulated between the conditions being compared. The complete data set is publicly available in the NCBI Gene Expression Omnibus (http://www.ncbi.nlm.nih.gov/geo/; accession numbers GSE11137).

### Quantitative real time RT-PCR

Total RNA was extracted using TRIzol, following manufacturer instructions. For reverse transcription, 60 ng of RNA were mixed with 5 uM random hexamers, 1 mM each dNTP, 7.5 mM MgCl_2_, 40 U RNasin (Promega, Madison, WI), 1X PCR buffer II (Applied Biosystems, Foster City, CA) and 250 U of SuperScript II reverse transcriptase (Invitrogen). The reaction mixture was incubated at 25°C for 10 min, 48°C for 45 min, and 95°C for 5 min, then cooled to 4°C. For reverse transcription, two volumes of ethanol were mixed with the cDNA product before transfer to a -20°C freezer for 30 minutes. The tubes were then centrifuged for 15 minutes at 16,000 X g, the pellets washed with 75% ethanol, and the product finally dried. The cDNA was re-suspended in distilled water and used as template for SYBR Green quantitative real-time PCR using primers designed for selected genes (Table S1). All primer pairs produced a single PCR product as determined by the dissociation curve and gel analysis. Real-time PCR was performed in a 20 ul reaction, 96-well format and 1 X SYBR PCR Master Mix (Applied Biosystems). Reaction plates were incubated in an Opticon 2 real-time PCR machine (MJ Research) for 40 cycles consisting of denaturation at 95°C for 15 s and annealing/extension at 58–60°C for 1 min. Three biological repeats at each development stage were analyzed in quadruplicate, with a minimum of two independent experiments. The relative amount of target mRNA normalized to GAPDH was calculated [28]. 

### Western blot analysis

Western blots were performed with rabbit anti-MARPs (kindly supplied by Siegfried Labeit), RBM20 (Home-made), Fhl1 (Santa Cruz), and anti-GAPDH (Santa Cruz). Total protein was separated by sodium dodecyl sulfate-polyacrylamide gel electrophoresis (SDS-PAGE) and transferred onto polyvinylidene difluoride (PVDF) membrane. Membrane was first blocked in phosphate-buffered saline-0.05% Tween 20 (PBST) with 5% nonfat dry milk overnight and then incubated with primary antibody diluted in blocking buffer for 3 hours at room temperature. After 3 washes with PBST, the membrane was incubated with horseradish peroxidase conjugated secondary antibody in PBST with 5% nonfat dry milk for 2 hours. After another 3 washes, the blot were developed with ECL western blotting substrate (Pierce) and exposed to CL-Xposure film (Thermo Scientific). The same membrane was then stripped and re-probed with anti-GAPDH which serves as a control to show that the total protein loads in different lanes are similar.

### Statistical analysis

GraphPad prism software was used for statistical analysis. Results are expressed as means ± SEM or SD. Statistical significance between groups was determined using one way ANOVA with a Tukey post test, or a 2-tailed t-test for comparison of two groups. The significance level was P<0.05, P<0.01 and P<0.001.

## Results

### RBM20^-/-^ rats express more protein and mRNA for the titin N2BA isoform

**Figure 1:**
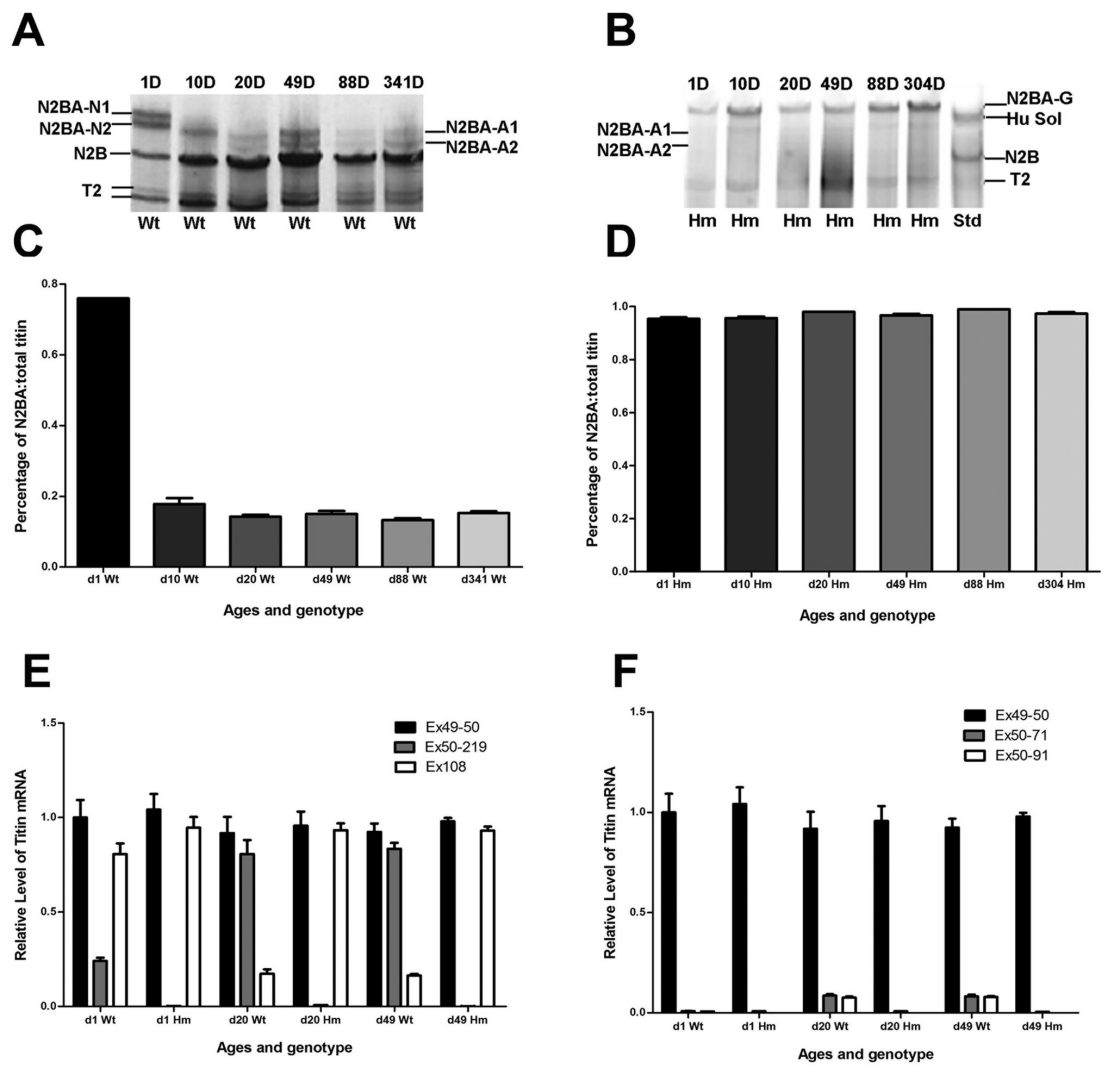
Developmental changes in expression of titin protein isoforms and mRNA in wild type (Wt) and homozygote (Hm) mutants. Proteins were separated by SDS agarose electrophoresis [16]. D: days after birth; Std: mixture of human soleus (3.7 MDa) & rat N2B (3.0 MDa); T2, titin fragment; Ex, exon. A. Larger N2BA isoforms (3.6 to 3.7 MDa) were present at one day after birth and disappeared with age in normal rats during development. B. A different larger N2BA isoform (3.8 MDa) appeared in mutant homozygote rats; the smaller N2B and other shorter N2BA isoforms were essentially absent at all ages. C. The smaller N2B isoform increased in proportion with age and reached approximately 85% (P<0.01) from 20 days of age and older in wild type. D. The larger N2BA isoform in homozygous mutants constituted nearly 100% (P<0.05) of the total titin. E. Relative titin mRNA expression in wild type and mutant rats determined by Q-PCR. All data are from an average of triplicates (P<0.05). Primers for exons 49 to 50 would prime both N2B and N2BA titin isoforms; primers for exons 50 and 219 would amplify the N2B only, and primers for exon 108 would amplify the N2BA isoforms only. F. Relative mRNA expression of other pre-identified titin isoforms to total titin in wild type and mutant rats. Total titin of 1 day Wt was defined as 1.0 for all the comparisons.

Titin normally undergoes a series of isoform expression changes with development as a result of alternative splicing (Figure 1A). The titin isoform protein proportions in wild type animals were ~25% N2B at day 1 after birth and ~85% N2B at day 20 and older rats (relative to total titin) respectively (Figure 1C). This developmental and adult expression pattern is disrupted when Rbm20 is absent [1,2]. Ventricle tissue from wild type, heterozygotes, and homozygote knockout rats had different agarose gel phenotypes at each time period after birth [2]. Homozygous knockouts expressed primarily a giant titin (~3.8 MDa) of the N2BA type at all ages examined (Figure 1B) [2,22] and the giant N2BA isoform constitutes almost 100% of total titin (Figure 1D). Quantitative real-time RT-PCR was performed to measure the level of titin isoform mRNAs in normal and knockout ventricular tissue at ages 1, 20, and 49 days (Figure 1E and F). Primer pair Ex49-50 (spanning exons 49 and 50) was designed to quantify total (N2B+N2BA) cardiac titin since these exons are constituently expressed in both isoform classes [8,9]. Wild type N2B mRNA level (primer pair Ex50-219 spanning exons 50 and 219) comprised approximately 24% of the total titin message at day 1 and 81-84% at days 20 and 49. However, titin N2B-mRNA was nearly undetectable in homozygote knockout rats at all stages examined. Wild type N2BA mRNA proportion (estimated using primers from exon 108) was approximately 81% at day 1 but dropped to 15-17% at the two later time points (Figure 1E). Total N2BA-mRNA is almost equal to total titin-mRNA in homozygous knockout rats at all three ages. These results are consistent with protein proportion of titin isoforms in the different genotypes and ages (Figure 1D). 

Quantitative estimates of two splice variants (Ex50-71 and Ex50-91) from the middle Ig region of the N2BA class (N2BA-A1 and N2BA-A2) were also measured (Figure 1F). Both Ex50-71 and Ex50-91 are almost undetectable in wild type hearts at day 1, but the Ex50-71 constituted about 8.5% of total titin message at day 20 and 49. Ex50-91 accounted for about 7.6% at day 20 and 49 in wild type rats. Homozygous knockout rats expressed only traces of the Ex50-71 and the Ex50-91 at all three ages, mirroring the lack of the corresponding intermediate sized titin protein isoforms (Figure 1C and D). 

### RBM20^-/-^ rats have reduced maximal exercise capacity

**Figure 2:**
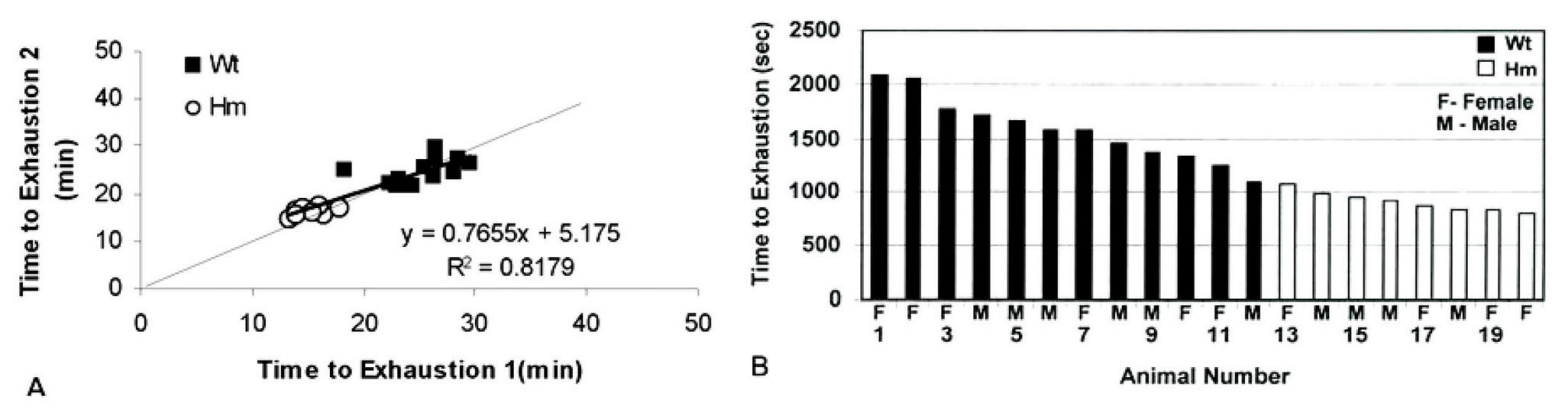
Exercise Capacity of wild type and mutant rats. Rats were exercised to exhaustion on a treadmill (see Materials and Methods). The running protocol was repeated twice. A. Reproducibility of measured maximal exercise capacity. The correlation of the 2 measurements was demonstrated by the large R^2^ value, near unity of slope, and small intercept; B. Wild type and homozygote mutant rats had significantly different maximal exercise capacity, defined as time to exhaustion. The maximal exercise capacity averaged 9.3 minutes longer in wild type rats than in mutants. P-value <0.001 was based on a T-test. Wt; wild type; Hm; Homozygous mutant; F; female; M; Male.

Maximal exercise capacity in wild type and homozygote knockout rats was used to assess the effect of the mutation on *in vivo* whole animal physiological function. Because the determination of exhaustion in a rat can be somewhat subjective and variable, we verified the reproducibility by conducting the maximal capacity test on each rat in duplicate. The correlation of the two measurements is demonstrated by a large R^2^ value, near unity of slope, and a small intercept. Figure 2A demonstrates not only very high reproducibility, but also clear stratification between the two groups. Maximal exercise capacity defined as time to exhaustion on a standardized treadmill running protocol was highly significantly lower in knockout rats (Figure 2B). The average of the maximal exercise capacity of the wild type is 9.3 minutes (p<0.001) longer than that of the homozygous knockout rats, and this was not due to differences in age, gender or body weight (Table S2 and Figure S1). 

### RBM20^-/-^ rat hearts have altered ultrastructure

**Figure 3:**
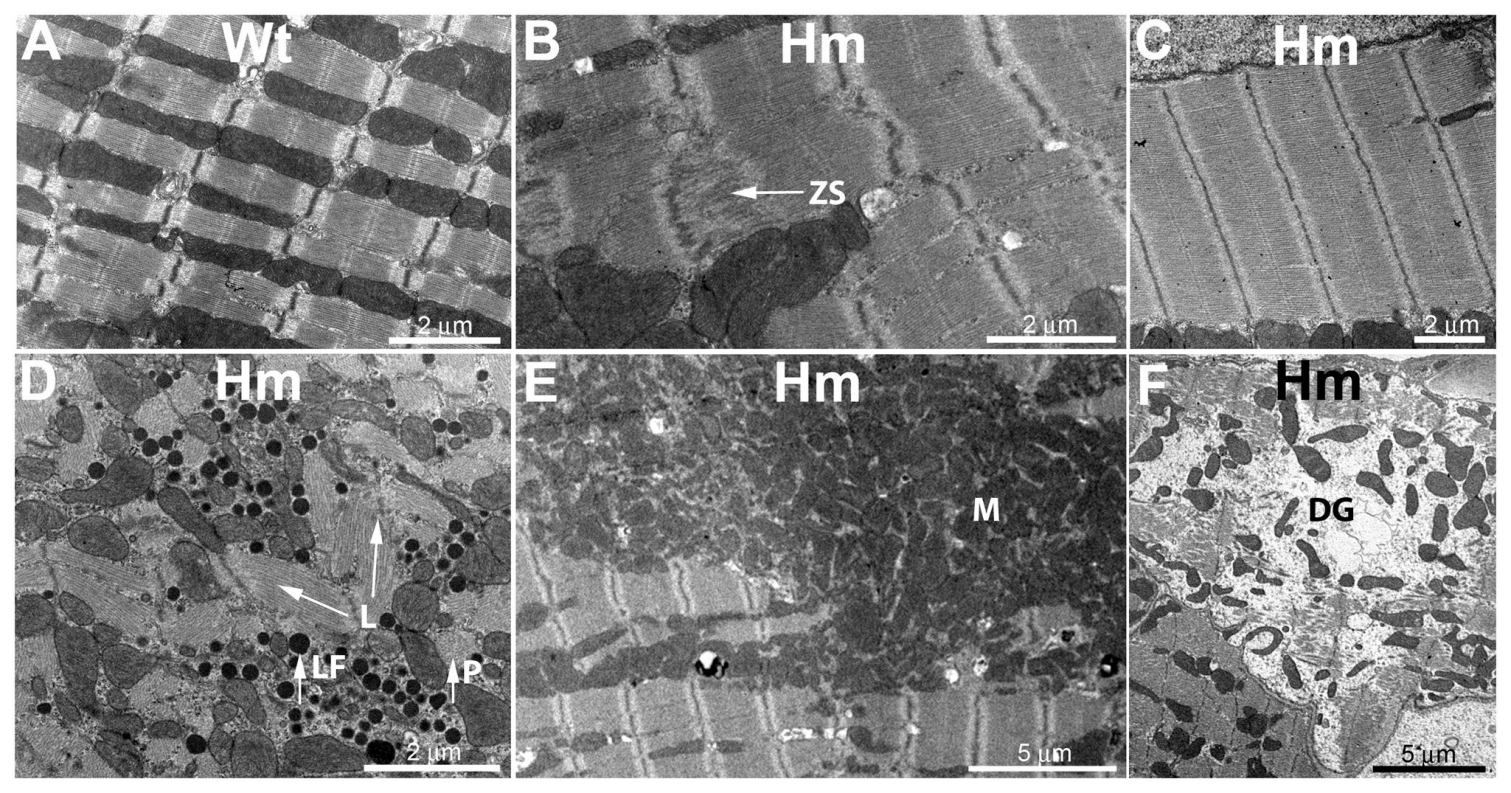
Electron microscopy of wild type and homozygous mutant left ventricle from rats one year of age. Samples were fixed, embedded, sectioned, and stained using standard methods prior to electron microscopic examination (see Materials and Methods for full details). A. Typical wild type cardiomyocyte appearance with myofibrils flanked by mitochondria. B. Homozygote with evidence of Z line streaming (arrow, ZS). C. Homozygote with exceptionally wide myofibril region. D. Homozygote with dark stained lipofuscin granules (arrow, LF). Filament bundles were observed with different orientations in the XY plane (arrows, L) and also running perpendicularly (arrow, P) E. Homozygote with a large cluster of mitochondria (M). F. Homozygote cardiomyocyte region showing myofibril disintegration (DG).

Titin functions as a template in sarcomere assembly and for maintenance of sarcomere integrity [29,30]. Thus changes in titin size might affect the sarcomeric structure and integrity. Samples from left ventricle of one year old wild type and homozygote knockouts were examined by electron microscopy. A typical example from a wild type heart is shown in Figure 3A. Myofibrils with well formed sarcomeres are visible, and these are interspersed regularly with mitochondria. Most areas of the homozygote knockout samples had a fairly normal appearance. However, a number of irregularities were observed that were never seen in age matched wild type hearts. These modifications included Z line streaming (ZS) (Figure 3B), lipofuchin granules (LF) and myofibril disarray[filaments both longitudinal (L) and perpendicular (P)] (Figure 3D), and regions of myofibril degeneration (DG) (Figure 3F). In addition a couple novel structural patterns were observed. Figure 3C shows an area with an extremely wide myofibril. Widths (perpendicular to the sarcomere longitudinal axis) exceeded 5 microns in several instances while widths greater than 2 microns were not seen with wild type hearts. Myocytes with these wide myofibril structures were often accompanied by a marked, irregular clustering of mitochondria (M) (Figure 3E). The appearance of the mutant mitochondria, however, was normal with membrane and cristae structure indistinguishable from wild type controls.

Electron microscopic observations of skeletal muscles from homozygote mutants were compared with those from wild type animals. Representative micrographs from tibialis anterior and longissimus dorsi are shown in Figure S2. The myofibril sarcomeres and filament lattice of the mutants were indistinguishable from wild type. Similarly there was no evidence of the mitochondrial clustering, wide sarcomeres, or degenerating regions seen in the mutant skeletal muscle samples. The only structural abnormalities observed in the mutants were a couple occurrences of Z line streaming, but the vast majority of the muscle regions appeared normal.

### RBM20^-/-^ rats have changes in heart failure related genes

**Figure 4:**
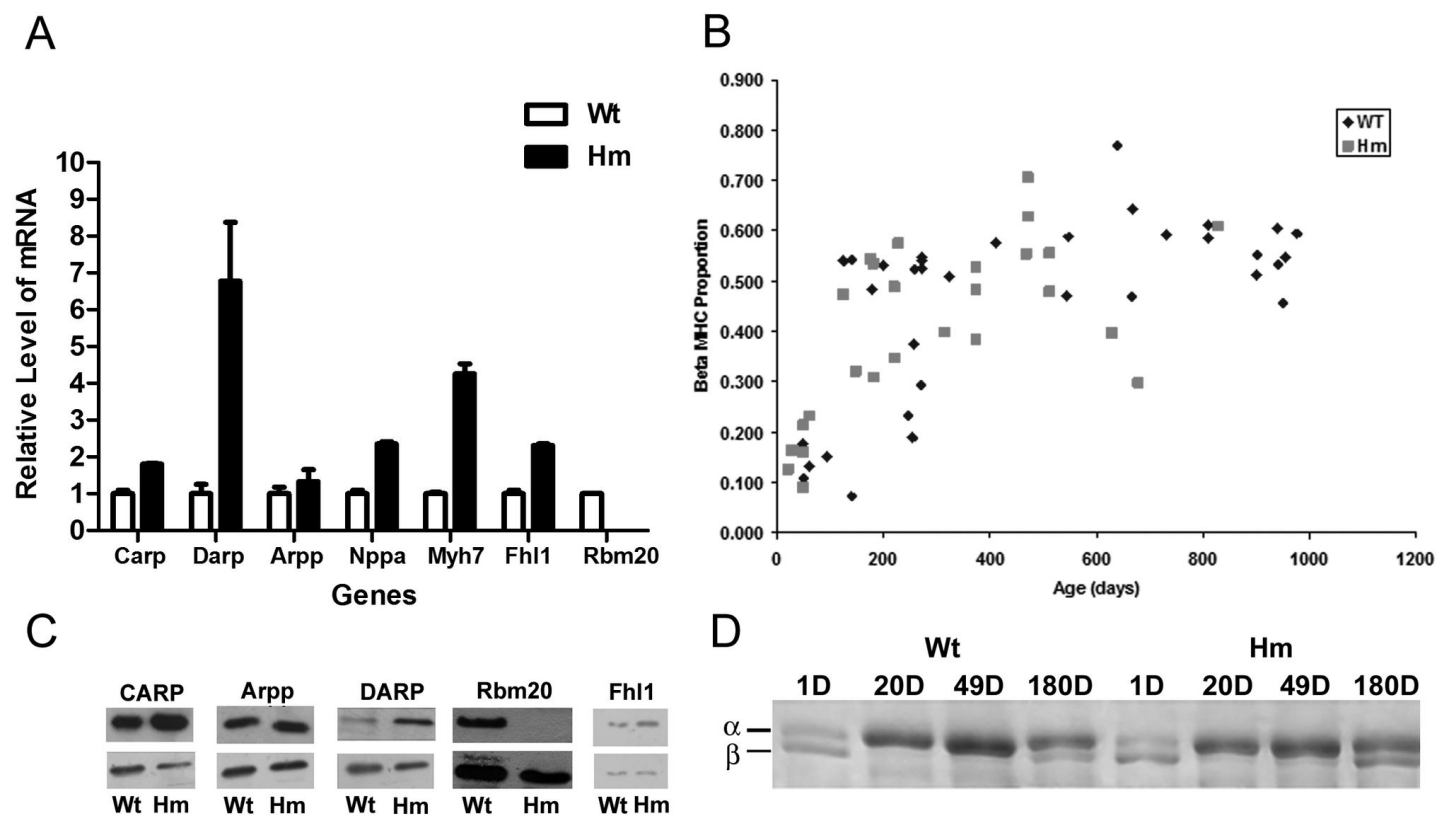
Expression levels of selected genes. A. Quantitative real time RT-PCR verification of microarray results for selected genes; wild type value set at 1.00 for each gene; all data are from an average of triplicates (P<0.05). C. Western blot analysis of MARPs and Rbm20. B and D. Left ventricle beta myosin heavy chain proportion and electrophoresis with various ages in the different phenotypes. D: day; Wt: wild type; Hm: Homozygote mutant.

We compared gene expression of homozygous knockout rat left ventricles versus wild type at 49 days of age using Affymetrix arrays. The 49 day age was selected as a time point where changes in titin splicing should be completed in wild type and potential secondary changes in the mutants would be limited. A total of 136 gene-identified transcripts were differentially up or down-regulated in knockout rat hearts (http://www.ncbi.nlm.nih.gov/geo/; accession numbers GSE11137)(Table S3) Among them, several muscle stress response and pathophysiological related genes (Table 1) were selected and verified with quantitative PCR measurements (Figure 4). CARP and DARP were significantly up-regulated at both the RNA and protein levels in knockout rats (Figure 4A and 4C). Arpp levels were not different between genotype groups. Rbm20 was undetectable at both the RNA and protein levels in knockout rats (Figure 4A and 4C), a finding consistent with our recent results [3]. Transcriptional level of beta myosin heavy chain increases in 49 day knockout rats detected by microarray (Table 1) and quantitative PCR (Figure 4A). However, there were no significant differences in the myosin heavy chain protein proportions of the different phenotypes at age 49 days or any other age up to > 2.7 years (Figure 4 B and D). 

**Table 1:** Selected differentially expressed genes in mutant rat left ventricle at 49 days of age.

Gene ID	Gene Symbol	Gene Description	Fold Change
64032	Ctgf	connective tissue growth factor	+2.48
81809	Tgfb2	Transforming growth factor, beta 2	+2.33
445442	Tsp1	thrombospondin 1	+2.05
27064	Ankrd1	Ankyrin repeat domain 1 (cardiac muscle)(CARP)	+1.60
316330	Ankrd23	ankyrin repeat domain 23 (DARP)	+3.68
25177	Fhl1	four and a half LIM domains 1	+2.58
29557	Myh7	myosin, heavy chain 7, cardiac muscle, beta	+3.92
24602	Nppa	natriuretic peptide precursor A	+3.10
309544	Rbm20	RNA binding motif protein 20	-20.36

Wild type values were set at 1.00.

## Discussion

Titin has been implicated as being responsible for passive tension, and other work has shown that the proportions of different sized titins affect passive tension [12,31,32]. The proportions are also altered with heart disease [14,15,21,33]. Previous studies with knockout cardiomyocytes expressing only the larger titin N2BA isoform showed they had markedly reduced passive tension [2]. Ca^++^- activated force development by skinned trabeculae was also reduced in the homozygote mutants [34]. Such changes might result in inadequate cardiac filling and/or in a smaller ejection fraction. These phenotypes have been observed with echocardiography in one and a half year old Rbm20^-/-^ animals [3]. Therefore, it is reasonable to speculate the Rbm20^-/-^rats might have a lower maximal cardiac output (i.e. impaired cardiac reserve), and the observation of reduced maximal exercise capacity in the Rbm20^-/-^ rats further supported this idea. Alternatively, maximal exercise capacity is determined by a complex, interacting set of factors including motivation, the ability of the lungs to oxygenate blood, and the ability of the skeletal muscles to extract and utilize oxygen for generation of ATP. Structurally, there appeared to be fewer electron microscopic abnormalities observed in mutant skeletal muscles than in cardiac. Other work from our laboratory has indicated that the protein profiles were essentially identical between wild type and mutant tibialis anterior [35]. Finally, the myosin heavy chain ratios of tibialis anterior and soleus were indistinguishable between wild type and mutants [36]. Thus we believe that the reduced exercise capacity of the mutants is due primarily to changes in the heart rather than in skeletal muscle. In the context of highly motivated otherwise healthy subjects, exercise capacity is often taken as a surrogate for maximal cardiac output. This can reflect inadequate cardiac filling, ejection or chronotropy, but titin’s putative role in the Frank-Starling response [29] and thus the exercise capacity remains to be determined. 

Heterozygous and homozygous Rbm20^-/-^ rat hearts had left ventricular dilatation, an increased percentage of sudden death, and increased fibrosis [3]. The electron microscopy results indicated that additional structural changes (Z line streaming, accumulation of lipofuscin granules, and myofibril disarray), and these phenotypes have been associated previously with human cardiac structural pathology [37-42]. Two additional unusual patterns were also observed – wide myofibrils (Figure 3C) and extensive mitochondrial clumping (Figure 3E). The former might be explained by the reduction in transverse forces due to the larger titin since such forces have been proposed as a mechanism for myofibril formation [43,44]. The mitochondrial grouping may be due to increased myofibril lysis within the Rbm20^-/-^ cardiomyocytes by an unknown mechanism. No ultrastructural evidence of changes in mitochondrial arrangement, density, or myofibril damage was observed in skeletal muscles from the homozygote mutants (Figure S2). 

The mechanisms that explain these alterations in muscle structure remain elusive. Since so many changes in gene isoform expression occur in the RBM20 mutant rats (the splicing of at least 29 genes are affected in addition to titin [3]), it would be pure speculation to ascribe them only to this latter protein. It is remarkable that the structural changes observed in these mutants have so many similarities to those observed in other species, including humans. 

Gene analysis indicated that disproportionality of titin isoform in cardiac muscle is consistent with a transcriptional change (measured by Q-PCR) to alter the isoform expression in the Rbm20^-/-^ rats. Message levels of N2B titin normally increase in the early neonatal period and those for N2BA decline as a proportion of the total titin message. The levels of message for the middle Ig splice variants (Ex50-71; Ex50-91) were consistent with the low levels of minor N2BA protein isoforms in wild types and virtual absence in the homozygous Rbm20^-/-^. The latter also had an essential absence of N2B message at all three ages examined. These results indicated that the Rbm20^-/-^ rats express almost exclusively the largest titin isoform (N2BA-G) [2], suggesting consistency between genotype and phenotype. 

Because titin isoform transition of 49 day old rats has been completed either with development in wild type or in the Rbm20^-/-^ rat [2,3], 49 day cardiac tissues were used for gene expression analysis. Transcriptional changes of global genomic expression demonstrated that CTGF, TGF-β, TSP1, Marps, Fhl1, Myh7, and Nppa are over-expressed in the Rbm20^-/-^ heart (Table 1). The first three genes interact in extracellular matrix (ECM) synthesis [45,46]. CTGF is an important mediator of TGF-β signaling in the heart and abnormal expression of this gene has been used as a diagnostic marker for cardiac fibrosis [47]. TSP1 can activate latent TGF-β by stimulation of growth factors such as Ang II and endothelin-1 (ET-1) [48,49]. Both CTGF and TSP1 are up regulated by TGF-β [50]. Furthermore, CTGF and TSP1 can promote the disassembly of focal adhesions and thus seem to be actively involved in tissue remodeling [51,52]. It should be noted that the up-regulation of these fibrosis-related genes is already present at 49 days of age even though the increase in trichrome positive staining material is not evident till rats are at least 3 months of age [3]. MARPs participate in muscle stress-activated pathways and are up-regulated in both cardiac and skeletal muscles after mechanical or metabolic challenge. Cyclic stretching of cultured cardiomyocytes induced expression of CARP and DARP both in the nucleus and in the sarcomeric I-bands [53], and end-stage failing human DCM hearts showed increased expression levels of MARPs [21,54]. Fhl1 interacts directly with the N2B region of titin to form a novel complex, and it has been proposed to act as a biomechanical sensor to myofibrillar passive tension generated upon stretch [55,56]. A dose-dependent increase in Fhl1 can reduce the titin N2B phosphorylation by interfering with the binding of Erk2 [57]. Therefore, the increase of Fhl1 in Rbm20^-/-^ rats is consistent with markedly reduced passive tension [2]. An increase of natriuretic peptide precursor A (Nppa), a blood pressure-dependent marker [58,59], suggests a supplementary mechanism to respond to (1) inadequate cardiac filling and ejection or (2) the increased cardiomyocyte size [2] in the Rbm20^-/-^ rats. Previous description on the up-regulation of these genes is consistent with the phenotype of the Rbm20^-/-^ rats that causes heart failure and signs of cardiomyopathy. These results suggest that the pathophysiological phenotype of the Rbm20^-/-^ rat results from changes in multiple genes triggered by modulating gene splicing [3]. Interestingly, almost all stress and heart failure related genes in the Rbm20^-/-^rat are up-regulated (Table 1). This could be due to adaptive response to change in stress signaling [54].

Previous studies showed that both 3,5,3'-triiodo-L-thyronine (T3) and insulin can restore or increase N2B proportion in cultured wild type neonatal cardiomyocytes [54,60]. A hypothyroidism rat model shows increased expression of a larger titin isoform [20]. Whether the Rbm20 is related to PI3K/AKT/mTOR signaling pathway or insulin signaling pathway remains to be determined. Finally, our previous results showed that there appeared to be no significant effect on the troponin T developmental time course in the Rbm20^-/-^ animals [2]. Although the myosin heavy chain β (Myh7) is up-regulated in mRNA level in knockout rat heart, there were no phenotype differences in protein levels between genotypes (Figure 4B, D). The left ventricle beta myosin heavy chain proportion increases with age in both knockout and wild type. 

In conclusion, altered splicing resulting from Rbm20 deficiency in our natural mutant rat model is related to the pathogenesis of cardiac muscle, but the mechanism can involve the regulation and interaction of multiple genes. The model should be invaluable for developing therapeutic treatments for the RBM20 human phenotype. 

## Supporting Information

Table S1 **Primers for quantitative real-time RT-PCR.**
(DOCX)

Table S2 **Animal phenotype data.** Sex and age paired animals were used for the running protocol. F: Female; M: Male; Wt: Wild type; Hm: Homozygote mutant.
(DOCX)

Table S3 **Left ventricle microarray comparisons of transcripts from wild type and homozygous Rbm20 knock out rats at 49 days of age.**
(DOCX)

Figure S1 **Animal phenotype data for running protocol.** A total of 12 wild type and 8 homozygote mutant rats were employed for running with matched gender and age within genotypes and between genotypes. *A* shows the matched age with standard deviation (P>0.05); *B* no weight differences between genotypes before and after running (P>0.05). Wt: Wild type; Hm: Homozygote mutant.
(DOCX)

Figure S2 **Electron microscopy of wild type and homozygous mutant skeletal muscle from rats one year of age.** A. Wild type tibialis anterior myofibril appearance. B. Homozygote tibialis anterior myofibril appearance. C. Wild type longissimus dorsi myofibril appearance. D. Homozygote longissimus dorsi myofibril appearance with a couple occurrences of Z line streaming.
(DOCX)
