# Supplementary material for: Pathophysiological Defects and Transcriptional Profiling in the RBM20-/- Rat Model
Source: PLoS One. 2013 Dec 19;8(12):e84281. doi: 10.1371/journal.pone.0084281 (PMC3868568; doi:10.1371/journal.pone.0084281)
Supplement: Table S1 — Primers for quantitative real-time RT-PCR. (DOCX) [file pone.0084281.s001.docx]

**Table S1**

| Exons | Sense primer(5'-3') | Antisense primer(5'-3') | Product size(bp) | Tm |
| --- | --- | --- | --- | --- |
| Ex50-219 | CAACGAGTATGGCAGTGTC | CTTTTCAGCACCACCTCCT | 129 | 60 |
| Ex49-50 | CTCAAACGGCAATGGGAAAG | CAGGTGAATTTGGCTAGGTG | 126 | 60 |
| Ex108 | GCATACAGAACATCGTGGTG | CTTTGTACCAGGTGACGATG | 91 | 60 |
| Ex50-71 | CCAACGAGTATGGCAGTGTCA | ACTACAGGCGGAAAGCTACTAAAAAC | 82 | 60 |
| Ex50-91 | CCTGCAAAGCCTCCAACG | GCTCCTTTAAGTGTCTCAAC | 101 | 60 |
| Ex71-80 | AGAACGAAGTGGGCAGTGAC | CTTATCTTGCATTCCAGTTGG | 128 | 60 |
| Ankrd1 | TGGAGAGTATGAAGCTGCTG | TCTGCCTCTCGAACTTTCTC | 116 | 60 |
| Arpp | GGACTTGCGACGTGAGATC | GCAGCTTTCAGGAATGTCTC | 167 | 60 |
| Ankrd3 | ACTTCATCAGCATTCAGCAG | TCAGGTTGTCCAGGGTCAG | 221 | 60 |
| Myh7 | GAGACAGAGAATGGCAAGAC | TCCTTGAGATTGTAGAGCAC | 137 | 60 |
| Rbm20 | CACTCCACCTTCCACATATC | GTCCCTTCTCCTGTAGCAC | 162 | 60 |
