# Supplementary material for: Pathophysiological Defects and Transcriptional Profiling in the RBM20-/- Rat Model
Source: PLoS One. 2013 Dec 19;8(12):e84281. doi: 10.1371/journal.pone.0084281 (PMC3868568; doi:10.1371/journal.pone.0084281)
Supplement: Table S2 — Animal phenotype data. Sex and age paired animals were used for the running protocol. F: Female; M: Male; Wt: Wild type; Hm: Homozygote mutant. (DOCX) [file pone.0084281.s002.docx]

**Table S2**

| **Animal Number** | **Gender** | **Genotype** | **Age (Day)** | **Weight(g) before running** | **Weight (g) after running** |
| --- | --- | --- | --- | --- | --- |
| **KE302** | F | Wt | 300 | 254.9 | 250.7 |
| **KE301** | F | Wt | 307 | 218.2 | 220 |
| **KE334** | F | Wt | 291 | 233.2 | 232.8 |
| **KE335** | F | Wt | 291 | 233.8 | 234 |
| **KE392** | F | Wt | 292 | 242.8 | 241 |
| **KE390** | F | Wt | 292 | 236.3 | 238 |
| **KE327** | F | Hm | 302 | 225.8 | 228 |
| **KE328** | F | Hm | 289 | 220.3 | 225.3 |
| **KE329** | F | Hm | 289 | 229.3 | 227.4 |
| **KE330** | F | Hm | 289 | 260.4 | 256.8 |
| **KE389** | M | Wt | 292 | 401.5 | 398.5 |
| **KE391** | M | Wt | 292 | 392.6 | 390.6 |
| **KE309** | M | Wt | 291 | 420.2 | 420 |
| **KE310** | M | Wt | 291 | 446.7 | 444 |
| **KE311** | M | Wt | 307 | 389.5 | 391.3 |
| **KE312** | M | Wt | 307 | 422.7 | 424.1 |
| **KE369** | M | Hm | 302 | 397.8 | 385.9 |
| **KE322** | M | Hm | 289 | 406.6 | 403.2 |
| **KE321** | M | Hm | 289 | 379.7 | 383.4 |
| **KE323** | M | Hm | 289 | 422.6 | 420.8 |
