# Supplementary material for: Pathophysiological Defects and Transcriptional Profiling in the RBM20-/- Rat Model
Source: PLoS One. 2013 Dec 19;8(12):e84281. doi: 10.1371/journal.pone.0084281 (PMC3868568; doi:10.1371/journal.pone.0084281)
Supplement: Table S3 — Left ventricle microarray comparisons of transcripts from wild type and homozygous Rbm20 knock out rats at 49 days of age. (DOCX) [file pone.0084281.s003.docx]

**Table S3**.

| **Gene Symbol** | **Description** | **Entrez Gene ID** | **Fold change** |
| --- | --- | --- | --- |
| Frmd3 | FERM domain containing 3 | 298141 | 12.67 |
| Csrp2 | cysteine and glycine-rich protein 2 | 29317 | 6.61 |
| Bex1 | brain expressed gene 1 | 501625 | 4.92 |
| Pde4dip | phosphodiesterase 4D interacting protein(Myomegalin) | 64183 | 4.36 |
| Myh7 | myosin, heavy chain 7, cardiac muscle, beta | 29557 | 3.92 |
| Fam160b1 | family with sequence similarity 160, member B1 | 361774 | 3.79 |
| Darp | ankyrin repeat domain 23 | 316330 | 3.68 |
| Penk1 | proenkephalin 1 | 29237 | 3.54 |
| Nppa | natriuretic peptide precursor A | 24602 | 3.10 |
| Casq1 | calsequestrin 1 (fast-twitch, skeletal muscle) | 686019 | 2.77 |
| Gnao | G protein, alpha activating activity polypeptide O | 50664 | 2.71 |
| RGD1306959 | similar to C11orf17 protein (predicted) | 361624 | 2.69 |
| Reg3g | regenerating islet-derived 3 gamma | 24620 | 2.67 |
| Mybpc2 | myosin binding protein C, fast-type | 292879 | 2.63 |
| Fhl1 | four and a half LIM domains 1 | 25177 | 2.58 |
| Ncam1 | Neural cell adhesion molecule 1 | 24586 | 2.55 |
| Ctgf | connective tissue growth factor | 64032 | 2.48 |
| Enah | enabled homolog (Drosophila) | 360891 | 2.46 |
| Znf23 | zinc finger protein 23 (KOX 16) | 307839 | 2.41 |
| Rrm2 | ribonucleotide reductase M2 | 362720 | 2.41 |
| Tgfb2 | Transforming growth factor, beta 2 | 81809 | 2.33 |
| Ildr2 | immunoglobulin-like domain containing receptor 2 | 685277 | 2.23 |
| Msrb2 | methionine sulfoxide reductase B2 | 361286 | 2.22 |
| Dnajc1 | DnaJ (Hsp40) homolog, subfamily C, member 1 | 291369 | 2.16 |
| Fam46a | family with sequence similarity 46, member A | 300870 | 2.07 |
| Cpa6 | carboxypeptidase A6 | 312913 | 2.07 |
| Tsp1 | thrombospondin 1 | 445442 | 2.05 |
| Dusp26 | dual specificity phosphatase 26 (putative) | 306527 | 2.04 |
| Syt3 | Synaptotagmin III | 25731 | 2.02 |
| Myot | myotilin | 291605 | 1.99 |
| Kcnq1 | potassium voltage-gated channel, subfamily Q, member 1 | 84020 | 1.95 |
| Tceal5 | transcription elongation factor A (SII)-like 5 | 680282 | 1.95 |
| Asb18 | ankyrin repeat and SOCS box-containing 18 | 316614 | 1.94 |
| Tnfrsf12a | tumor necrosis factor receptor superfamily, member 12a | 302965 | 1.90 |
| Thbp | crystallin, mu/ thyroid hormone binding protein (THBP), | 117024 | 1.88 |
| Tdrd1 | tudor domain containing 1 | 292129 | 1.86 |
| Cks2 | CDC28 protein kinase regulatory subunit 2 | 498709 | 1.83 |
| Cdkn3 | cyclin-dependent kinase inhibitor 3 | 289993 | 1.83 |
| Rnd1 | Rho family GTPase 1 | 362993 | 1.77 |
| Mllt11 | myeloid/lymphoid or mixed-lineage leukemia | 295264 | 1.76 |
| Wbp5 | WW domain binding protein 5 | 680354 | 1.75 |
| Hspb3 | heat shock protein 3 | 78951 | 1.75 |
| Septin8 | septin 8 | 303135 | 1.71 |
| Tmem29 | transmembrane protein 29 | 683034 | 1.69 |
| Slc35a2 | solute carrier family 35, member A2 | 100158233 | 1.68 |
| Hebp1 | heme binding protein 1 | 362454 | 1.68 |
| Hsf2 | Heat shock factor 2 | 64441 | 1.68 |
| Map1b | microtubule-associated protein 1B | 29456 | 1.67 |
| Eif4e2 | eukaryotic translation initiation factor 4E member 2 | 363275 | 1.65 |
| Adamts19 | ADAM metallopeptidase with thrombospondin type 1 motif, 19 | 361332 | 1.63 |
| Lrrc16a | leucine rich repeat containing 16A | 306941 | 1.62 |
| Cdc2a | cell division cycle 2, G1 to S and G2 to M | 54237 | 1.61 |
| Carp | Ankyrin repeat domain 1 (cardiac muscle) | 27064 | 1.60 |
| LOC688274 | hypothetical protein LOC688274 |  | 1.59 |
| Gstm5 | glutathione S-transferase, mu 5 | 64352 | 1.57 |
| LOC680230 | hypothetical protein LOC680230 | 680230 | 1.57 |
| Tox3 | TOX high mobility group box family member 3 | 291908 | 1.56 |
| Flywch1 | FLYWCH-type zinc finger 1 | 360488 | 1.55 |
| Dusp8 | Dual specificity phosphatase 8 | 361679 | 1.53 |
| Popdc3 | Popeye domain-containing 3 | 641520 | 1.52 |
| LOC683034 | hypothetical protein LOC683034 | 683034 | 1.52 |
| Sypl2 | synaptophysin-like 2 | 362018 | 1.51 |
| Asb5 | Similar to ankyrin repeat and SOCs box-containing protein 5 | 361187 | 1.51 |
| Cenpk | centromere protein K | 294712 | 1.50 |
| Btbd11 | BTB (POZ) domain containing 11 | 314675 | -1.50 |
| Kcnj11 | potassium inwardly rectifying channel, subfamily J, member 11 | 83535 | -1.50 |
| Polr1a | polymerase (RNA) I polypeptide A | 83581 | -1.51 |
| Kcnd3 | potassium voltage gated channel, Shal-related family, member 3 | 65195 | -1.51 |
| Lpin1 | Lipin 1 | 313977 | -1.51 |
| RGD1304963 | Similar to hypothetical protein MGC38716 | 313891 | -1.51 |
| Gamt | guanidinoacetate N-methyltransferase | 25257 | -1.51 |
| Coq10a | coenzyme Q10 homolog A (S. cerevisiae) | 362810 | -1.51 |
| Bche | butyrylcholinesterase | 65036 | -1.52 |
| Asrgl1 | asparaginase like 1 | 246307 | -1.52 |
| Acss2 | acyl-CoA synthetase short-chain family member 2 | 311569 | -1.52 |
| Rt1-A2 | RT1 class Ia, locus A2 | 24974 | -1.52 |
| Fndc5 | fibronectin type III domain containing 5 | 260327 | -1.54 |
| Ptgds | prostaglandin D2 synthase (brain) | 25526 | -1.54 |
| Gstz1 | glutathione transferase zeta 1 | 681913 | -1.55 |
| Sts | steroid sulfatase | 24800 | -1.56 |
| Rxrg | retinoid X receptor gamma | 83574 | -1.56 |
| Dhrs7c | dehydrogenase/reductase (SDR family) member 7C | 287411 | -1.56 |
| Ttc39a | tetratricopeptide repeat domain 39A | 298366 | -1.57 |
| LOC681849 | similar to Protein C6orf142 homolog | 681849 | -1.57 |
| Lipg | lipase, endothelial | 291437 | -1.58 |
| Rragd | Ras-related GTP binding D | 297960 | -1.58 |
| Ldhd | lactate dehydrogenase D | 307858 | -1.58 |
| Ddit4l | DNA-damage-inducible transcript 4-like | 140582 | -1.58 |
| Atcay | ataxia, cerebellar, Cayman type | 362826 | -1.60 |
| Rtn2 | reticulon 2 | 308410 | -1.60 |
| Cadps | Ca++-dependent secretion activator | 26989 | -1.61 |
| Gpd2 | glycerol-3-phosphate dehydrogenase 2, mitochondrial | 25062 | -1.61 |
| Gngt2 | G protein, gamma transducing activity polypeptide 2 | 690825 | -1.61 |
| Myo5b | myosin Vb | 25132 | -1.61 |
| Fam81a | family with sequence similarity 81, member A | 315789 | -1.62 |
| Alkbh2 | alkB, alkylation repair homolog 2 (E. coli) | 304578 | -1.62 |
| Creb3l2 | cAMP responsive element binding protein 3-like 2 | 362339 | -1.63 |
| Ank2 | ankyrin 2, neuronal | 362036 | -1.63 |
| Fam100b | family with sequence similarity 100, member B | 287840 | -1.63 |
| Lmod3 | leiomodin 3 (fetal) | 500267 | -1.63 |
| Pgam2 | phosphoglycerate mutase 2 (muscle) | 24959 | -1.64 |
| Slc4a3 | solute carrier family 4 (anion exchanger), member 3 | 24781 | -1.64 |
| Gpt1 | glutamic-pyruvate transaminase (alanine aminotransferase) | 81670 | -1.64 |
| Klf9 | Kruppel-like factor 9 | 117560 | -1.66 |
| Cpd | Carboxypeptidase D | 25306 | -1.67 |
| Ptprr | protein tyrosine phosphatase, receptor type, R | 94202 | -1.68 |
| Plin5 | perilipin 5 | 501283 | -1.71 |
| Masp1 | mannan-binding lectin serine peptidase 1 | 64023 | -1.73 |
| Auts2 | Autism susceptibility candidate 2 (predicted) | 304419 | -1.74 |
| Caprin1 | cell cycle associated protein 1, transcript variant 2 | 362173 | -1.74 |
| Pla2g2d | phospholipase A2, group IID | 298579 | -1.75 |
| Mccc1 | methylrotonoyl-coenzyme A carboxylase1 | 294972 | -1.78 |
| Agpat2 | 1-acylglycerol-3-phosphate O-acyltransferase 2 | 311821 | -1.78 |
| Slc22a23 | solute carrier family 22, member 23 | 64559 | -1.79 |
| Sfxn5 | sideroflexin 5 | 261737 | -1.81 |
| LOC689926 | hypothetical protein LOC689926 | 689926 | -1.82 |
| Kcnd2 | potassium voltage gated channel, Shal-related family, member 2 | 65180 | -1.83 |
| Tnfaip8 | tumor necrosis factor, alpha-induced protein 8 | 307428 | -1.84 |
| Card9 | caspase recruitment domain family, member 9 | 64171 | -1.88 |
| LOC498368 | similar to RIKEN cDNA 0610040J01 | 498368 | -1.89 |
| Cds1 | CDP-diacylglycerol synthase 1 | 81925 | -1.90 |
| Sgms2 | sphingomyelin synthase 2 | 310849 | -1.91 |
| Rbm32b | RNA binding motif protein 32B | 302405 | -2.03 |
| Cox7aH | Cytochrome c oxidase polypeptide VIIa-heart | 687508 | -2.04 |
| Cxcl11 | chemokine (C-X-C motif) ligand 11 | 305236 | -2.12 |
| Acsl6 | acyl-CoA synthetase long-chain family member 6 | 117243 | -2.29 |
| Kcnk2 | potassium channel, subfamily K, member 2 | 170899 | -2.35 |
| LOC682679 | hypothetical protein LOC682679 | 682679 | -2.55 |
| LOC298139 | similar to RIKEN cDNA 2310003M01 | 298139 | -2.98 |
| Hs3st5 | heparan sulfate (glucosamine) 3-O-sulfotransferase 5 | 294449 | -3.07 |
| Ngdn | Neuroguidin, EIF4E binding protein | 305887 | -3.44 |
| Trdn | Triadin | 59299 | -3.92 |
| Ablim1 | actin-binding LIM protein 1 | 307989 | -3.95 |
| Sctr | secretin receptor | 81779 | -3.96 |
| Scn4b | sodium channel, type IV, beta | 315611 | -5.26 |
| Rbm20 | RNA binding motif protein 20 | 309544 | -20.36 |
