# Supplementary figures and images for: Pathophysiological Defects and Transcriptional Profiling in the RBM20-/- Rat Model
Source: PLoS One. 2013 Dec 19;8(12):e84281. doi: 10.1371/journal.pone.0084281 (PMC3868568; doi:10.1371/journal.pone.0084281)

**Figure S1**


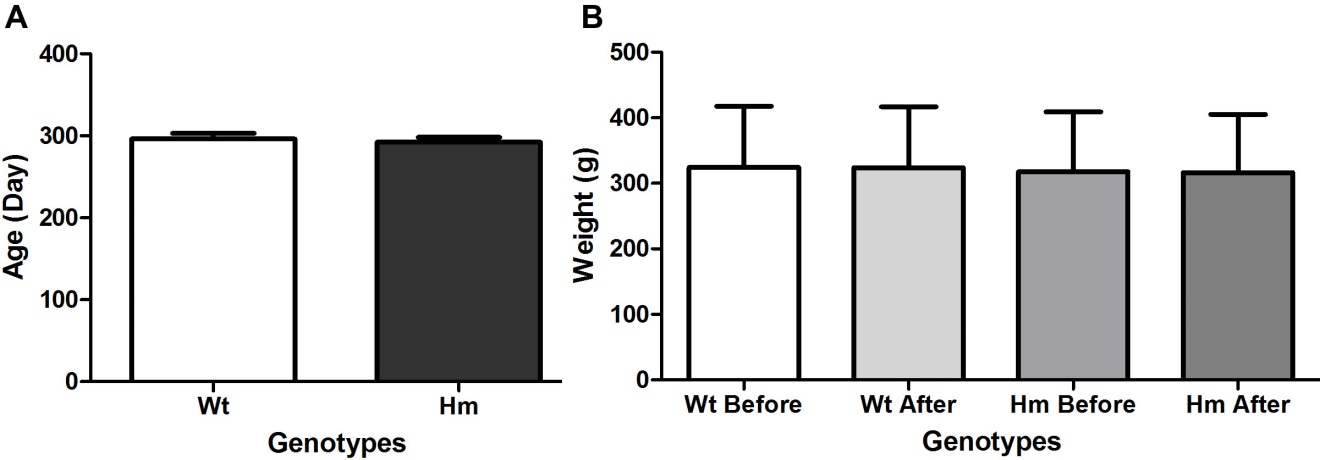

Supplement: Figure S1 — Animal phenotype data for running protocol. A total of 12 wild type and 8 homozygote mutant rats were employed for running with matched gender and age within genotypes and between genotypes. A shows the matched age with standard deviation (P>0.05); B no weight differences between genotypes before and after running (P>0.05). Wt: Wild type; Hm: Homozygote mutant. (DOCX) [file pone.0084281.s004.docx]

Figure S2:


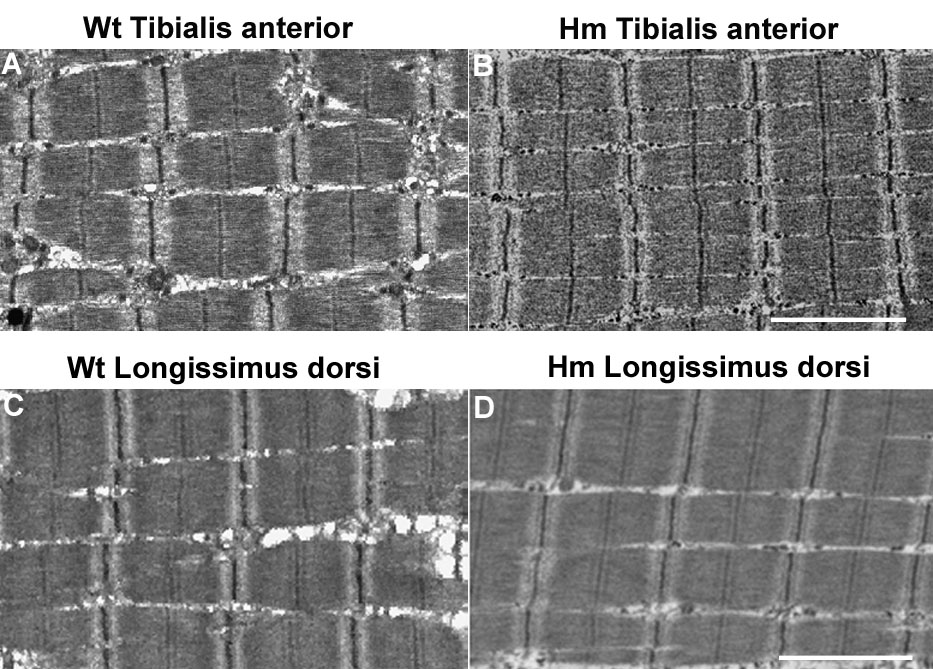

Supplement: Figure S2 — Electron microscopy of wild type and homozygous mutant skeletal muscle from rats one year of age. A. Wild type tibialis anterior myofibril appearance. B. Homozygote tibialis anterior myofibril appearance. C. Wild type longissimus dorsi myofibril appearance. D. Homozygote longissimus dorsi myofibril appearance with a couple occurrences of Z line streaming. (DOCX) [file pone.0084281.s005.docx]
